# Breaking Barriers: Exploring New Treatment Approaches for Major Depressive Disorder

**DOI:** 10.1192/j.eurpsy.2026.11405

**Published:** 2026-07-31

**Authors:** T. T. Pieters, A. Herremans

**Affiliations:** Pharmaceutical Sciences, Utrecht University, Utrecht, Netherlands

## Abstract

**Introduction:**

Depression and anxiety account for an estimated 12 billion lost workdays each year, costing the global economy approximately $1 trillion annually. Despite extensive research, the past 30 years have yielded only limited improvements in the effectiveness of treatments for Major Depressive Disorder (MDD). Numerous scientific disciplines continue to explore potential therapies, yet no approach has delivered a transformative breakthrough. Current efforts at interdisciplinary collaboration remain insufficient, leaving patients in urgent need of more effective solutions.

**Objectives:**

The primary objective is to foster transdisciplinary collaboration among researchers, clinicians, and patients in order to overcome structural and cultural barriers that limit progress. The ultimate goal is to accelerate the development of effective multimodal treatments that significantly improve the lives of individuals living with MDD.

**Methods:**

A critical analysis was conducted to identify the systemic obstacles that hinder collaboration. Key barriers include communication gaps between disciplines, entrenched academic silos, competitive rather than cooperative mindsets, and funding structures that reward that reward interdisciplinary over transdisciplinary collaboration. As countermeasures, the initiative proposes to:

Center the patient perspective to strengthen awareness of the need for collaboration.

Create dedicated spaces—such as patient society events, and healthcare gatherings—for transdisciplinary dialogue.

Identify and integrate currently absent disciplines and patient organizations into existing conversations.

**Results:**

While radical systemic change is beyond immediate scope, initial steps demonstrate that raising awareness and fostering transdisciplinary dialogue are achievable entry points. The way forward includes developing a shared proposition, visualizing problems and solutions (van Baalen et al. Front Psychiatry 2025;15:1463929.), engaging patient societies, and cultivating positive motivation across stakeholders. Together, these steps can break down barriers and lay the groundwork for a transformative paradigm shift in MDD treatment.

**Image 1:**
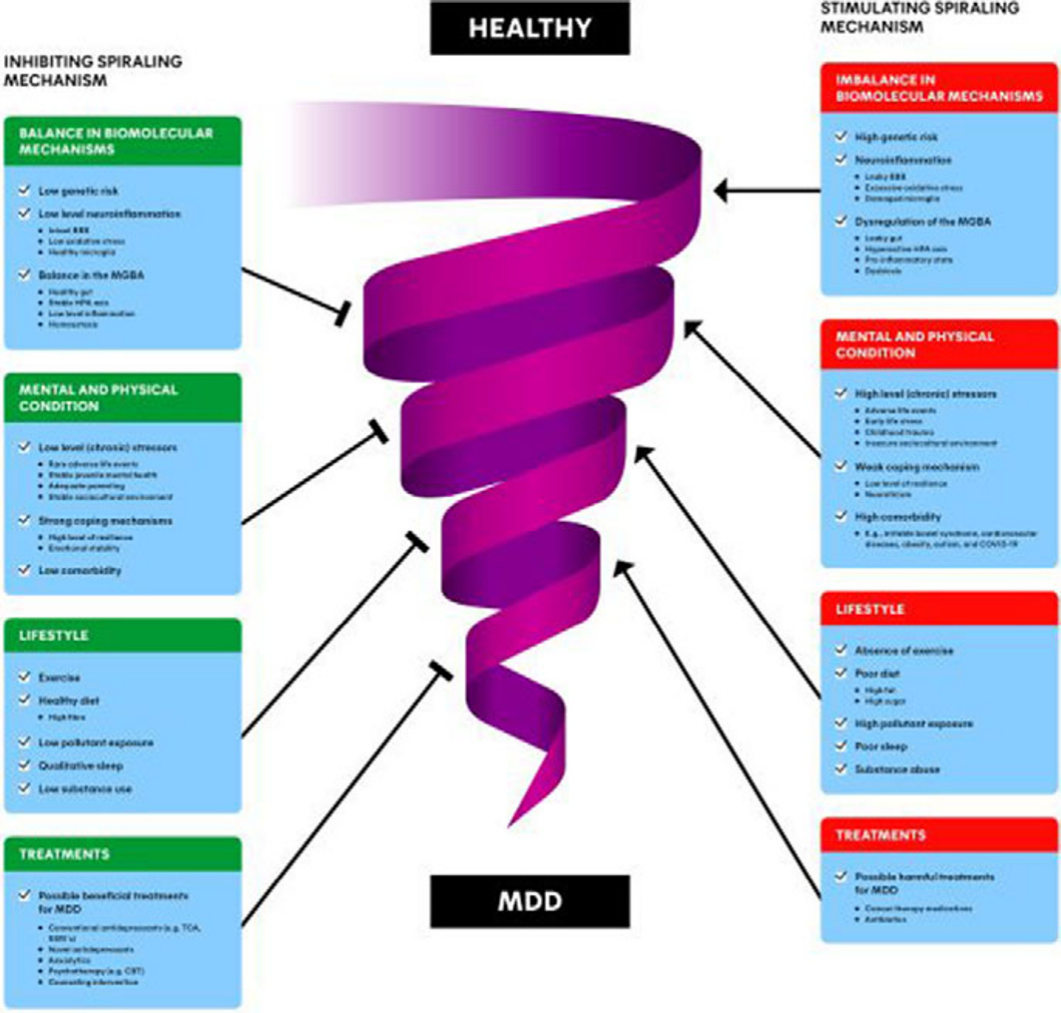
Image 1: Long description.

**Conclusions:**

By fostering awareness and creating opportunities for transdisciplinary dialogue, the groundwork is laid for a paradigm shift in MDD treatment, which brings tangible benefits to patients and redefines the future of care.

**Disclosure of Interest:**

None Declared

## Long descriptions

### Image 1: Long description

A central purple ribbon spirals downward, narrowing from a top label marked HEALTHY to a bottom label marked M D D.

On the left side, under the heading INHIBITING SPIRALING MECHANISM, four green-capped blue boxes point inhibitory T-bar arrows toward the spiral.

• BALANCE IN BIOMOLECULAR MECHANISMS: includes low genetic risk, low level neuroinflammation (intact B B B, low oxidative stress, healthy microglia), and balance in the M G B A (healthy gut, stable H P A axis, low level inflammation, homeostasis).

• MENTAL AND PHYSICAL CONDITION: includes low level chronic stressors, strong coping mechanisms (high level of resilience, emotional stability), and low comorbidity.

• LIFESTYLE: includes exercise, healthy diet (high fiber), low pollutant exposure, qualitative sleep, and low substance use.

• TREATMENTS: includes possible beneficial treatments for M D D such as conventional antidepressants (T C A, S S R I), novel antidepressants, anxiolytics, psychotherapy (C B T), and counseling intervention.

On the right side, under the heading STIMULATING SPIRALING MECHANISM, four red-capped blue boxes point active arrows toward the spiral.

• IMBALANCE IN BIOMOLECULAR MECHANISMS: includes high genetic risk, neuroinflammation (leaky B B B, excessive oxidative stress, damaged microglia), and dysregulation of the M G B A (leaky gut, hyperactive H P A axis, pro-inflammatory state, dysbiosis).

• MENTAL AND PHYSICAL CONDITION: includes high level chronic stressors (adverse life events, early life stress, childhood trauma, insecure sociocultural environment), weak coping mechanism (low level of resilience, neuroticism), and high comorbidity (I B S, cardiovascular disease, obesity, autism, and C O V I D 19).

• LIFESTYLE: includes absence of exercise, poor diet (high fat, high sugar), high pollutant exposure, poor sleep, and substance abuse.

• TREATMENTS: includes possible harmful treatments for M D D such as cancer therapy medications and antibiotics.

Navigate back to Image 1:.

